# Clinical characteristics and detection of MYB-QKI fusions in patients with angiocentric glioma

**DOI:** 10.1007/s10072-024-07721-3

**Published:** 2024-08-05

**Authors:** Tiemin Li, Adilijiang Aihemaitiniyazi, Huawei Zhang, Da Wei, Yue Hu, Yuguang Guan, Jian Zhou, Xueling Qi, Mengyang Wang, Bin Wu, Mingwang Zhu, Linpeng Zhang, Guoming Luan, Changqing Liu

**Affiliations:** 1 Department of Neurosurgery, Sanbo Brain Hospital, Capital Medical University, No. 50, Yikesong, Xiangshan, Haidian District, Beijing, 100093 China; 2 Department of Neurosurgery, Beijing Chaoyang Hospital, Capital Medical University, Beijing, 100020 China; 3 Department of Neurosurgery, Aviation General Hospital, China Medical University, Beijing, 100012 China; 4 Department of Pathology, Sanbo Brain Hospital, Capital Medical University, Beijing, 100093 China; 5 Department of Neurology, Sanbo Brain Hospital, Capital Medical University, Beijing, 100093 China; 6 Department of Radiology, Sanbo Brain Hospital, Capital Medical University, Beijing, 100093 China

**Keywords:** Angiocentric glioma, Clinical characteristics, MYB-QKI, Tumor

## Abstract

**Purpose:**

Angiocentric glioma (AG), a benign tumor identified within the last two decades, was officially included in the 2007 WHO Classification of Tumors of the Central Nervous System, WHO grade I. The tumor is relatively rare, with only approximately 100 cases reported. We aim to complement the characteristics and long-term prognosis of AG, as well as to detect MYB-QKI fusions.

**Methods:**

The characteristics of all cases collected between 1 March 2009 and 1 March 2023 at the Beijing Sanbo Brain Hospital, Capital Medical University, were summarized and analyzed. Additionally, all fourteen patients were tested for MYB-QKI fusions.

**Results:**

AG more predominantly occurs in adolescents (median age 16.5-year-old), and commonly presents with drug-resistant epilepsy. AG is frequently localized in the supratentorial regions and only one patient is in the brainstem. Brain parenchyma atrophy, and stalk-like signs can observe in imaging. Pathologically, tumor cells are perivascular pseudorosettes, presenting immunoreactivity for GFAP, S-100, Vimentin, “dot-like” staining for EMA, and low proliferative activity. Focal cortex dysplasia was observed in four patients. Twelve of fourteen (85.7%) patients were found with MYB-QKI fusions. Completely surgical resection typically has a satisfactory prognosis with long-term follow-up.

**Conclusion:**

AG is a rare benign tumor with a favorable prognosis after complete resection, characterized by refractory epilepsy, frequently occurring in adolescents. MYB-QKI fusions were detected in most AG patients, as a good defining genetic alteration pathologically. The potential presence of focal cortical dysplasia (FCD) may affect the prognosis of epilepsy.

**Supplementary Information:**

The online version contains supplementary material available at 10.1007/s10072-024-07721-3.

## Introduction

Angiocentric glioma (AG) is a rare low-grade glioma. Wang et al. described such a tumor as angiocentric bipolar astrocytoma in 2002. Wang and Lellouch-Tubiana first characterized systematically and pathologically that the tumor was vascular-centered and arranged in a pseudorosettes pattern in 2005. Immunohistochemistry extrapolated from GFAP immunoreactivity and EMA-positive and ultrastructural characteristics, the tumor exhibited glial and ependymal differentiation and was described as monomorphous angiocentric glioma [1, 2]. In 2007, Preusser et al. identified it as an angiocentric neuroepithelial tumor based on positive staining for glial fibrillin, S-100 protein, and chromophilic granulin [3]. In 2007, the World Health Organization designated angiocentric glioma as WHO grade I due to its slow growth, rare postoperative recurrence, benign biological behavior, and low proliferation index [4]. However, AG falls into the “other neuroepithelial tumors” category, along with chordoid glioma and astroblastoma, due to its unclear tissue origin [5]. The latest 5th edition of the World Health Organization’s Central Nervous System Cancer Classification for 2021 classifies AG as a “pediatric diffuse low-grade glioma“ [6].

Most AG affect adolescents, and most patients begin with drug-resistant epilepsy. AG is common in the supratentorial region and mostly involved a single lobe of brain. Similar magnetic resonance imaging (MRI) characteristics are exhibited in several AG cases, such as peritumor edema, brain parenchyma atrophy and stalk-like signs (defined as T2WI or FLAIR hyperintensity that tapers toward the lateral ventricle) [7]. The main criteria for AG diagnosis include a perivascular growth pattern, features of monomorphous bipolar cells with positive of GFAP, S-100, Vimentin and “dot-like” staining for epithelial membrane antigen (EMA). AG is clinically rare, with nearly 100 cases reported in the literature. We herein report 14 additional cases of AG, furthermore, we detected the MYB-QKI fusions of all patients.

## Materials and methods

### Materials

According to the 2021 WHO classification of Tumors of the Central Nervous System, all patients who underwent surgical resection of occupied space were diagnosed with “angiocentric glioma” between March 1, 2009, and March 1, 2023, at Beijing Sanbo Brain Hospital, the Capital Medical University. All patients were Asians born in China. Clinical, imaging, and pathologic data were collected from the Beijing Sanbo Brain Hospital medical record system.

### Methods

The data from 14 pathologically diagnosed AG were collected, and their general clinical, imaging, and pathologic features were summarized. To verify the possibility of the existence of focal cortical dysplasia in epilepsy-related tumors, we selected two additional patients. The pathology and imaging of two patients with focal dysplasia were compared.

The brain tissue specimens that were surgically removed from the patients were fixed with 3.7% neutral formaldehyde, embedded in conventional paraffin, sliced into 5-µm thick sections, and then stained with hematoxylin and eosin (H&E). Immunohistochemical staining was performed with the following primary antibodies: glial fibrillary acidic protein (GFAP; Dako, polyclonal, dilution 1:1000), vimentin (Zymed; monoclonal, dilution 1:200), epithelial cell membrane antigen (EMA; Zymed, monoclonal, dilution 1:100), oligodendrocyte transcription factor 2 (Olig-2; Immuno-Biological Laboratories, polyclonal, dilution 1:500), neuronal nuclear antigen (NeuN; Chemicon, monoclonal, dilution 1:2000), CD34 (Zymed, monoclonal, clone QBEnd 10, dilution 1:50), isocitrate dehydrogenase (IDH1R132H; Zymed, monoclonal, dilution 1:1000), Ki-67 (MIB-1; OriGene, monoclonal, clone UMAB107, 1:200), BRAFV600E (Spring Bioscience, monoclonal, clone VE1, dilution 1:50), synaptophysin (Biogenics, polyclonal, dilution 1:50).

Further MYB-QKI fusions gene detection was achieved by Fluorescence in situ hybridization (FISH) dual-color probes (QKI probe, green fluorochrome, MYB probe, red fluorochrome, both from Abbott, B190304). FISH analysis was performed on 2.5 ∼ 3 μm sections prepared from archived formalin-fixed and paraffin-embedded (FFPE) materials. The sections were pre-treated in 75 °C oven for 60 min, deparaffinized in xylene/isopropanol, and pressure cooked in citrate buffer for 2 min. Following digestion in a coplin jar with 4 mg/ml pepsin at 37 °C for 3–20 min, the sections were hybridized with the denatured probes at 37 °C for 10 ∼ 18 h. Then the slides were washed with 2XSSC and counterstained. Hybridization signals were analyzed using an Olympus fluorescence microscope equipped with applied imaging software. Two experienced pathologists independently reviewed the FISH images and observed more than 200 cells, the positive result was defined as the proportion of cells with MYB-QKI fusion genes was greater than 15% and interpreted the results.

## Results

### Clinical information

The clinic data of the fourteen cases are summarized in Supplementary Table 1. Nine of the fourteen AG patients were male, while five were female. The median age at diagnosis was 16.5 years (range, 4–58 years). Eight out of fourteen patients were under 18 years old, while the other six were over 18 years old. Eleven patients had epilepsy (11/14, 78.6%), two had paroxysmal headaches (case 5 and case 11), and one had dizziness (case 14, the lesion was in the pons). Among the eleven patients presenting with epilepsy as their clinical manifestation, one patient exhibited simple partial seizure (SPS), three exhibited complex partial seizure (CPS), two exhibited generalized tonic-clonic seizure (GTCS), four exhibited both CPS and GTCS, and one exhibited both SPS and GTCS. The illness duration ranged from three days to 22 years, with seven patients experiencing a duration of more than one year and seven patients experiencing less than one year; six patients took two or more antiepileptic drugs (AEDs) orally, but the symptoms did not improve, other patients did not take two or more AEDs because they were not epileptic or had a short duration of disease. Only three patients had pre-existing conditions (two had a history of febrile seizures, and one had difficulty giving birth).

### Imaging

**Fig. 1:**
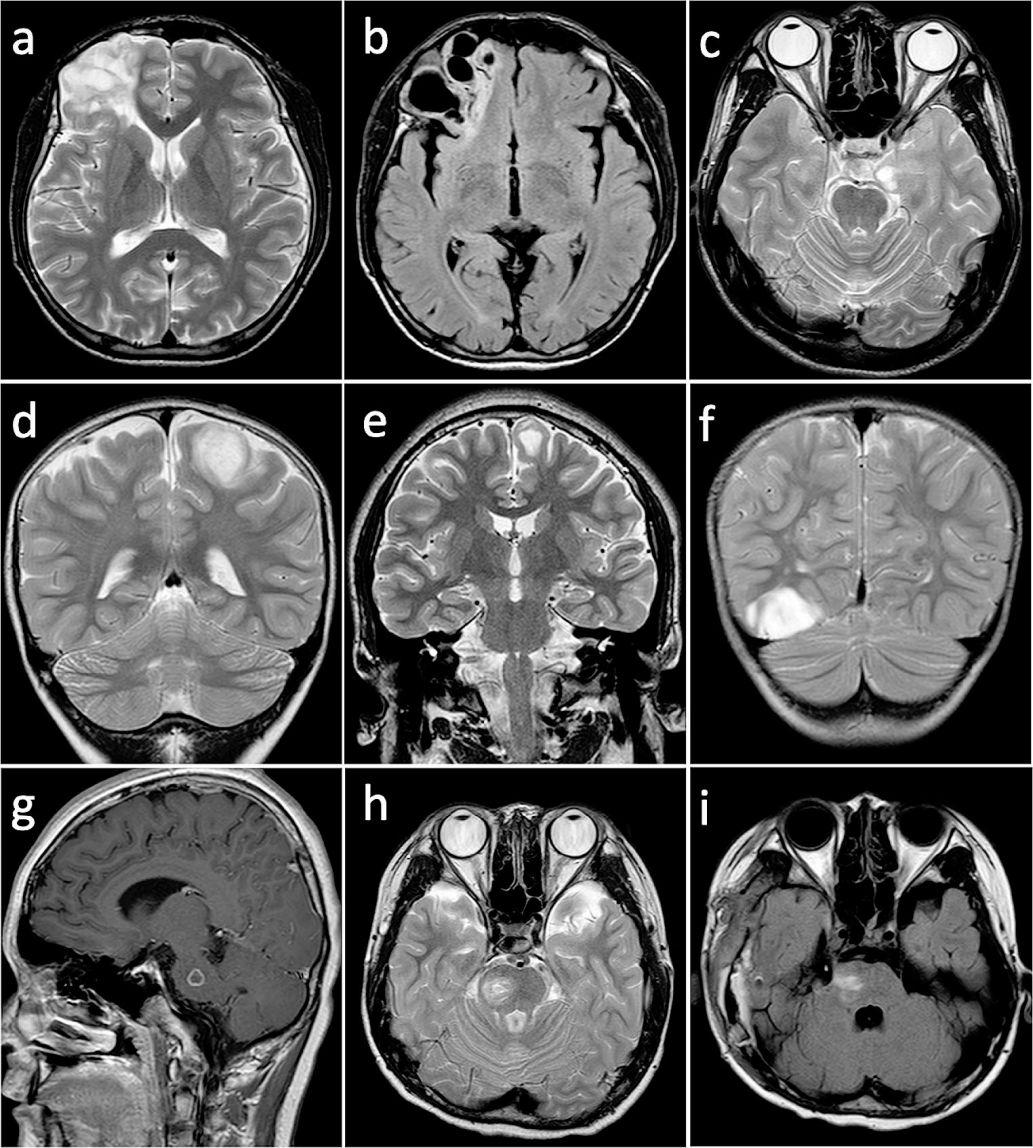
**a**,** b.** Case 1, MRI showed a polycystic lesion with high T2-weight signal and rim-like high FLAIR signal in the right frontal lobe; **c.** Case 2, T2-weighted image showed high signal in the left mesial temporal lobe; **d**,** e**,**f.** Case 9,10,11, stalk-like signs and brain parenchyma atrophy can be observed; **g**,** h**,**i.** Case 14, lesion was observed in the right side of pons

The imaging data for fourteen patients is summarized in Table 1. Among the fourteen AG patients, tumors were located on the right side in seven cases and on the left side in another seven. Thirteen patients had supratentorial tumors, and among these patients, two had tumors involving multiple cerebral lobes, while only one patient had an infratentorial tumor located in the pons. The radiological features of the tumors were described as patchy and lobulated. There were two cases presenting as circular, two as cystic solid, one as spindle, and one as a mass. In Case 3, the patient’s tumor had indistinct borders; the others were well-defined. Most patients’ tumors had a maximum diameter of less than 2 cm (11/14, 78.6%), while the remaining had a diameter greater than 2 cm (3/14, 21.4%). Among the fourteen patients, MRI revealed enhanced signals in five patients (5/14, 35.7%). Twelve patients, including two with solid cystic lesions, showed low to slightly low signals on T1-weighted images and equal high signals on T2-weighted images, with high signals on Fluid-attenuated inversion recovery (FLAIR) images (case 1 showed rim-like high signal, Fig. 1a, b), slightly higher signal intensity.

**Table 1:** Imaging data of the fourteen cases

Case	Laterality/Location	Tumor morphology/ margin	Tumor size(mm)	T1/T2/FLAIR-weighted image	Contrast enhancement	Peritumoral edema	Brain parenchyma atrophy	Stalk-like sign
1	Right/ Frontal, insular, parietal lobe	Lobulated/ Well	53 × 35 × 34	Low High High(rim-like)	No	Yes	Yes	Yes
2	Left/ Temporal lobe	Patchy/ Well	14 × 10 × 14	Low High High(rim-like)	No	No	No	No
3	Right/ Temporal lobe	Patchy/ Not well	30 × 20 × 20	Low High High	No	NA	NA	NA
4	Left/ Frontal lobe	Cystic solid/ Well	21 × 19 × 36	Low Equal/High High	Yes	NA	NA	NA
5	Right/ Temporal lobe	Circular/ Well	49 × 30 × 40	Low High High	Yes	NA	NA	NA
6	Left/ Temporal, occipital lobe	Patchy/ Well	40 × 45 × 45	Low High Slightly high	No	NA	NA	NA
7	Left/ Parietal lobe	Spindle/ Well	40 × 40 × 30	Low High High	Yes	NA	NA	NA
8	Left/ Temporal lobe	Mass/ Well	52 × 11 × 37	Low High Slightly high	Yes	NA	NA	NA
9	Left/ Frontal lobe	Circular/ Well	21 × 15 × 25	Low High Slightly high	No	Yes	Yes	Yes
10	Left/ Frontal lobe	Patchy/ Well	21 × 15 × 16	Low High Slightly high	No	Yes	No	Yes
11	Right/ Parietal lobe	Cystic solid/ Well	20 × 14 × 14	Low High High	Yes	NA	NA	NA
12	Right/ Occipital lobe	Patchy/ Well	31 × 30 × 22	Low High High	No	Yes	Yes	Yes
13	Right/ Temporal lobe	Patchy/ Well	45 × 45 × 45	Slightly low High Slightly high	No	NA	NA	NA
14	Right/ Pons	Patchy/ Well	18 × 18 × 18	Slightly low High High	No	Yes	No	No

Stalk-like sign: defined as T2WI or FLAIR hyperintensity that tapers toward the lateral ventricle

NA: not available

Only six of the fourteen patients collected imaging data (excluding data loss and extramural imaging data). Within the MRI findings for these patients, peritumoral edema was present in five cases, various degrees of brain parenchymal atrophy in three, and a stalk-like sign in four. Peritumoral edema and cerebral atrophy were more frequently observed on the MRI of patients with larger tumors and those presenting with a stalk-like sign (Fig. 1a-b, d-e).

### Electrophysiology

Six patients (all with epilepsy as their primary symptom) underwent more complete neurophysiological examinations, including video-electroencephalogram (v-EEG), magnetoencephalogram (MEG), and electrocorticography (ECoG). The examination revealed a spectrum of epileptic activities among the six patients: focal discharges were detected in two, diffuse discharges in another two, and multifocal discharges in one patient, while another showed no detectable discharges. The detected discharges’ locations were consistent with lesion sites identified through imaging. This concordance underscores the reliability of EEG and MEG in pinpointing epileptic activity.

### Pathology

**Fig. 2:**
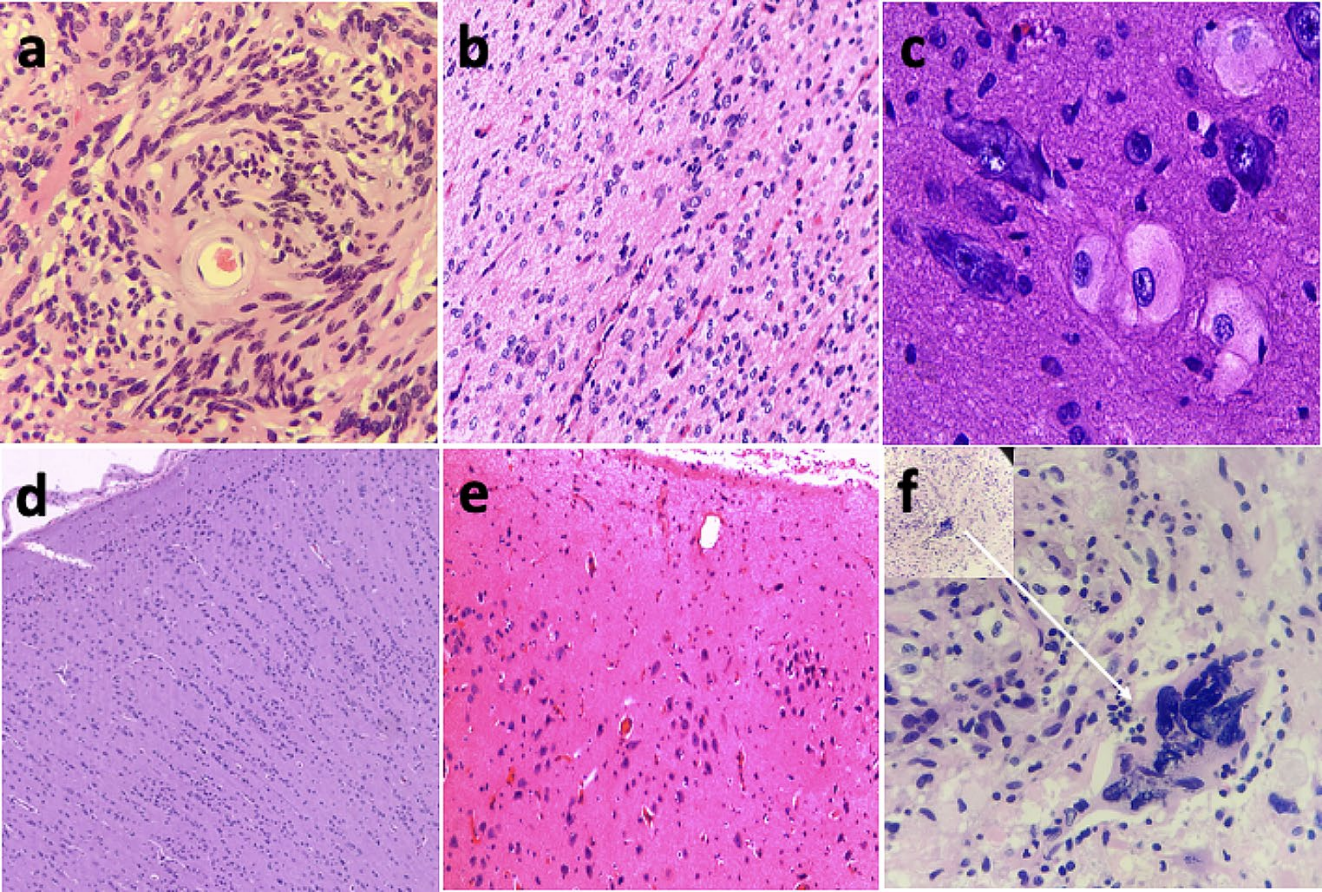
**a.** Case 10, tumor cells vascularized and arranged in a perivascular pseudorosettes pattern; **b.e.** Case 6 ,10, both cases demonstrated cortical tangential abnormalities adjacent to the tumors; **c.** Case 7, dysmorphic neurons and balloon cells were observed; **d.** Case 8, this case demonstrated cortical radial abnormalities; **f.** Case 7, pleomorphic xanthoastrocytoma (PXA) tumor-like and tumor giant cells observed in one patient

The immunohistochemistry data of the fourteen cases are summarized in Table 2. All fourteen patients showed similar histopathological and immunohistochemical characteristics. The tumor cells were spindle-shaped, bipolar, single-layered, or multi-layered, arranged in a perivascular pseudorosettes pattern and ependymal-like structure (Fig. 2a). Peripheral glia and small blood vessel hyperplasia were observed in some cases. The tumor tissue was partially calcified in three patients. Additionally, evidence of FCD was observed in four cases (Fig. 2b-e). Case 6 and Case 10 exhibited cortical tangential abnormalities adjacent to the tumors, which were ultimately classified as FCD IIIb. In Case 7, dysmorphic neurons and balloon cells were observed in the cortex surrounding the tumor, which was consequently classified as FCD IIb. Case 8 demonstrated cortical radial abnormalities and was finally classified as FCD Ia. Pleomorphic xanthoastrocytoma (PXA) tumor-like and tumor giant cells were observed in case 7 (Fig. 2f).

**Table 2:** Immunohistochemistry and cytogenetics of the fourteen cases

Case	GFAP	S-100	Vimentin	EMA	Olig-2	NeuN	CD34	IDI-1	IDH-1	Ki-67	BRAF	P53	D2-40	MGMT	MYB-QKI fusions
1	+	+	+		+(weak)	+(weak)				<1%		-		+	+
2	+	+	+	+	+(weak)		+(vas)			<1%					+
3	+	+	+	+	-	+	+(vas)			<1%					+
4	+	+	+	-	+		-		-	1%		+(weak)		+	+
5	+	+	+	+(weak)	+(weak)	+(weak)	+(vas)		-	<1%		+(weak)	-	-	-
6	+	+	-	-/+	+	+	+(vas)		-	<1%		+(weak)	-		+
7	+	+	+	+	+	+(weak)	+(vas)		-	3%		+(weak)		-	+
8	+	+(weak)	+	-/+	+	+	+(vas)	+	-	3%		+(weak)	+	+(weak)	+
9	+	+(weak)	+	+	+(weak)			+	-	2%		+	+	+	+
10	+	+	+	+	+(weak)	-	-	+	-	2%		+(weak)		+(weak)	+
11	+	+(weak)	+		+(weak)	-		+		1%	-/+	+(weak)		+	-
12	+	+	+	+(weak)	+(weak)	-	-	+	-	3 ∼ 5%(local)	+	+(weak)		+	+
13	+(weak)	+	+	+	-	-	-	+	-	<1%		-		+	+
14	+	+	+	+	-	-	-	+	-	1 ∼ 2%		-	+	+	+

+=positive, -=negative

+(weak) = few or partial or individual or local positive

+(vas) = vascular positive

GFAP and S-100 were positive in 14 patients, with a positivity rate of 92.8% (13/14) for Vimentin, 92.3% (12/13) for EMA, 78.6% (11/14) for Olig-2, and 54.5% (6/11) for NeuN and CD34. IDI-1 was 100% positive (7/7), while IDH-1 was negative (10/10). Ki-67 was a low expression, less than 5% (Supplementary Fig. 1). BRAF was analyzed in two patients, one showed positive (case 12) and one showed equivocal or borderline positive (case 11).

### Follow-up

Fourteen patients were followed at different times, ranging from 3.8 to 13.2 years. Most patients whose seizures began were on antiepileptic drugs for 1–3 years, and one patient had a local EEG review two years after surgery revealed ongoing brain discharge. All patients did not experience tumor or seizure recurrence after surgery, including one patient who experienced occasional dizziness. The remaining three patients did not exhibit a recurrence of corresponding symptoms after the operation.

### Imaging versus pathology

One patient (Supplementary Fig. 2a) was a 20-year-old man with 5 years duration of absence seizures and simple partial seizures. MRI showed a slightly high signal in the inferior part of the right temporal lobe on FLAIR-weighted. The anterior temporal lobe (5.5 × 4 × 3 cm) and the head and body of hippocampus (4 × 2 × 2 cm) were removed surgically. Histopathological examination showed mild cortical dysplasia in temporal lobe and neuronal and mixed neuronal-glial tumors in hippocampus. Another patient (Supplementary Fig. 2b) was a 5-year-old boy with 1 month duration of absence seizures and complex partial seizures. MRI showed a slightly high signal in the left frontal lobe on FLAIR-weighted. The tumor was accepted wide excision (3 × 3 × 2 cm), and pathology showed focal cortical dysplasia, type IIb.

We analyzed pathologic samples from two epilepsy patients with FCD included in the pathologic diagnosis near the lesion using MRI. We observed partial loss of normal cortical structure, abnormal arrangement of cortical nerve cells, and abnormal morphology of nerve cells around the lesions in both cases (Supplementary Fig. 2a-b).

### MYB-QKI fusions gene detection

All fourteen patients were screened for MYB-QKI fusions. The findings revealed that twelve out of the fourteen patients exhibited MYB-QKI fusions. Notably, no positive result was observed in Case 5 and Case 11 (Supplementary Fig. 3).

## Discussion

As reported in previous research studies, AG primarily affects adolescents, with a higher proportion of male patients. The youngest reported case is 1.5 years old, and while cases in the elderly are less common, the oldest reported patient is 83 years old [8, 9]. AG is frequently slow growing and has a longer course of disease, with nearly half of patients having a course of more than two years and most adults having a course of more than 10 years [10]. Approximately 90% of patients begin with drug-resistant epilepsy and begin with a long history of multiple refractory epilepsy. Others may have vision loss, dizziness, eye pain, ataxia, dysphagia, weakness in limbs, and more [11–13]. Covington et al. reported an AG patient in the midbrain whose gait was characterized by obstructive hydrocephalus [14], whereas Rosenzweig et al. reported a patient with auditory hallucinations but considered epilepsy-related [15]. Zhang et al. reported a case with acute intracranial hemorrhage as the first symptom [16]. In our cases, 78.6% (11/14) patients exhibited epilepsy as their primary manifestation, consistent with findings from prior studies. Nonetheless, there were instances of patients presenting with alternative clinical features, two patients with paroxysmal headaches had elevated intracranial pressure due to intracranial space-occupying lesion. one patient had dizziness but no seizures, which are associated with occupying the pons, where seizures frequently involve the cortex.

AG is most common in the supratentorial region, and primarily in the frontal, temporal, and parietal lobes, less common in the occipital lobes, cerebral islands, thalamus, hippocampus [17, 18], and a few in the brainstem [13, 14, 19, 20]. AG typically does not occur outside the skull, however, one patient had an angiocentric glioma in the spinal cord due to extracranial metastasis [21]. Most of the AG forms were solid [22], and a few reported cystic changes with solid forms [23]. Certain features of AG imaging may exist, including the high density of the T1-weight of the tumor visible on MRI images, the high signal phase of the T2Flair-weight extending to the ventricle, known as the “stalk-like sign,” and regional cerebral atrophy. Kurokawa et al. suggested that intratumoral T1-weighted high-intensity areas were significantly more prevalent in patients exhibiting stalk-like signs and associated regional atrophy, those characteristic imaging manifestations of AG may be associated with prolonged seizures, co-occurring FCD, and glial hyperplasia associated with the inert and tumor nature [7]. However, not all AG patients described here exhibit these features, and previous reports indicate that the presence of these three characteristics does not necessarily confirm AG. One patient had these characteristics but was eventually diagnosed with ependymoma [24], indicating that imaging features are an inaccurate diagnostic basis for AG but rather a tool to aid diagnosis.

Currently, AG diagnosis is based on pathologic and immunohistochemical results. The primary feature is that single bipolar spindle cells are arranged around cortical blood vessels or neurons in concentric sleeves and pseudorosettes, with some cases exhibiting cortical dysplasia or calcification foci [11]. Immunohistochemical staining results are typically positive for GFAP, S-100, and vimentin, with EMA exhibiting a dot-like pattern [23, 25]. The Ki-67 proliferative index was usually < 5%, but in malignant transformation or extracranial metastasis cases, the proliferation index can be ≥ 25% [21, 26]. In our cases, we discovered one case of AG with pleomorphic xanthoastrocytoma(PXA) tumor-like regions, a finding similar to previous reports of AG presenting with oligodendroglioma tumor-like regions [27] or astroblastoma-like characteristics [28]. As the gold standard for diagnosis, AG patients exhibit common features in pathology and immunohistochemistry. However, with the anticipated discovery of an increasing number of AG cases demonstrating aggressive tendencies and anaplastic characteristics in the future, adjustments to diagnostic criteria may be necessitated.

Studies have demonstrated that abnormalities in AG genomes primarily affect the v-myb avian myeloma virus oncogenic homolog (MYB) gene located at 6q23.3, where MYB genes are normally fused to form the MYB-QKI fusion protein, which is observed in most AG tumor cells [19, 20, 29–34]. MYB immunohistochemistry is a sensitive marker for these tumors, displaying a strong and diffuse expression [25]. However, local MYB expression was detected in several other low-grade gliomas [32]. In our cases, there were two patients with AG who did not show the MYB-QKI rearrangement, these patients may exhibit a distinct genetic alteration involving MYB but not QKI, as mentioned in the 2021 WHO classification [6]. Interestingly, these two patients had the clinical manifestation of paroxysmal headaches rather than epilepsy. This discovery suggests the presence of another subgroup of AG patients with a different molecular profile and potentially different clinical outcomes. Further research is needed to understand the underlying mechanisms and implications of this genetic alteration in AG pathogenesis. Additionally, it highlights the importance of comprehensive genetic profiling in the diagnosis and management of AG, as different molecular subtypes may require tailored therapeutic approaches.

Treatment with AG is typically surgical, with a low risk of recurrence after complete tumor resection. In most patients, treatment with anti-epileptic drugs over time after complete tumor resection essentially controls seizures. After a puncture biopsy identifies the pathology, radiotherapy is feasible in patients who cannot be surgically resected, thereby reducing tumor size and relieving symptoms [3, 8]. AG is defined as WHO grade I, with a low risk of malignancy, but there have been cases of recurrence or invasion [2, 26, 31, 35, 36], with only one patient having extracranial metastases [21] and one with partially resected AG dying from tumor progression [2]. It is important to note that although the majority of AG are benign, due to their characteristic presentation with epilepsy, electroencephalography with video monitoring and magnetoencephalography are utilized to delineate the extent of the epileptogenic focus. During surgery, ECoG is employed to identify potential epileptogenic zones surrounding the tumor while ensuring its complete removal. The surgical field is further expanded based on these findings, and post-resection ECoG monitoring confirms the absence of spike waves. Currently, few cases of AG with FCD have been reported. Liu et al. reported three cases of AG with FCD, suggesting that the extent of epileptogenic focus is not limited to the tumor itself and that FCD may be a factor in AG’s uncontrolled seizures after complete resection [37]. In the 14 cases included, pathological examination revealed four cases with concurrent focal cortical dysplasia. Interestingly, three out of these four individuals underwent comprehensive preoperative and intraoperative electrophysiological monitoring, suggesting that the application of electrophysiological examinations may facilitate a more complete resection of the epileptogenic focus in AG patients, potentially influencing their prognosis. Therefore, the simple removal of the occupying lesion is insufficient for tumor-associated epilepsy patients, the extent of lesion resection should be based on neuro-electrophysiological confirmation of the lesion. Additionally, one patient had occasional postoperative dizziness, which may be related to the extent of the tumor.

## Conclusions

AG is a benign tumor of the central nervous system that commonly affects adolescents. Clinically, it typically presents with epilepsy, and often resistant to pharmacological treatment. It is more frequently localized in the supratentorial regions and characterized by low T1-weighted, high T2-weighted, and high FLAIR-weighted signals on imaging. Some cases may demonstrate T1-weighted enhancement. The definitive diagnosis is usually confirmed through histological and immunohistochemical analyses post-surgical excision. However, for AG patients presenting clinically with epilepsy, it may be challenging to distinguish, on imaging, between the angiocentric glioma lesion and potential surrounding focal cortical dysplasia. Moreover, as epileptogenic zone is often not limited to the tumor and may be reside in the areas of focal cortical dysplasia and within the tumor, the presence of focal cortical dysplasia may impact the prognosis. Therefore, to prevent recurrence of epilepsy, it is necessary to simultaneously remove tissues affected in both areas. However, no large-scale data has been reported due to the rarity of clinical cases of AG.

## Electronic supplementary material

Below is the link to the electronic supplementary material.

Supplementary Material 1
