# Supplementary material for: Clinical characteristics and detection of MYB-QKI fusions in patients with angiocentric glioma
Source: Neurol Sci. 2024 Aug 5;46(1):427–36. doi: 10.1007/s10072-024-07721-3 (PMC11698799; doi:10.1007/s10072-024-07721-3)
Supplement: Supplementary file 1 — Supplementary Material 1 [file 10072_2024_7721_MOESM1_ESM.docx]

**Supplementary Table 1．**Clinic data of the fourteen cases

| Case | Sex | Diagnosis age（yrs） | Symptom | Duration | First Treatment | Past History | Follow-up（yrs） | Outcomes |
| --- | --- | --- | --- | --- | --- | --- | --- | --- |
|  |  |  |  |  |  |  |  |  |
| 1 | M | 26 | CPS+GTCS | 22y | AEDs | Difficulty giving birth | 13.2 | Good |
| 2 | M | 23 | CPS+GTCS | 14y | AEDs | Febrile seizures | 12.5 | Good |
| 3 | M | 15 | CPS | 2y | AEDs |  | 11.7 | Good |
| 4 | F | 17 | CPS | 1m |  |  | 9.9 | Good |
| 5 | F | 29 | Paroxysmal headaches | 4y |  |  | 9.5 | Good |
| 6 | F | 7 | GTCS | 6y | AEDs | Febrile seizures | 9.4 | Good |
| 7 | F | 16 | GTCS | 1w |  |  | 9 | Good |
| 8 | M | 43 | CPS+GTCS | 15y | AEDs |  | 9 | Good |
| 9 | F | 9 | CPS | 0.5y |  |  | 8.1 | Good |
| 10 | M | 20 | CPS+GTCS | 13y | AEDs |  | 8.1 | Good |
| 11 | M | 15 | Paroxysmal headaches | 3d |  |  | 7 | Good |
| 12 | M | 4 | SPS+GTCS | 2w | AED |  | 7 | Good |
| 13 | M | 58 | SPS | 1w |  |  | 4.6 | Good |
| 14 | M | 12 | Dizziness | 3m |  |  | 3.8 | Good |

SPS=simple partial seizure

CPS=complex partial seizure

GTCS=generalized tonic-clonic seizure

AEDs=anti-epileptic drugs

Good=Seizure-free and no recurrence of the tumor


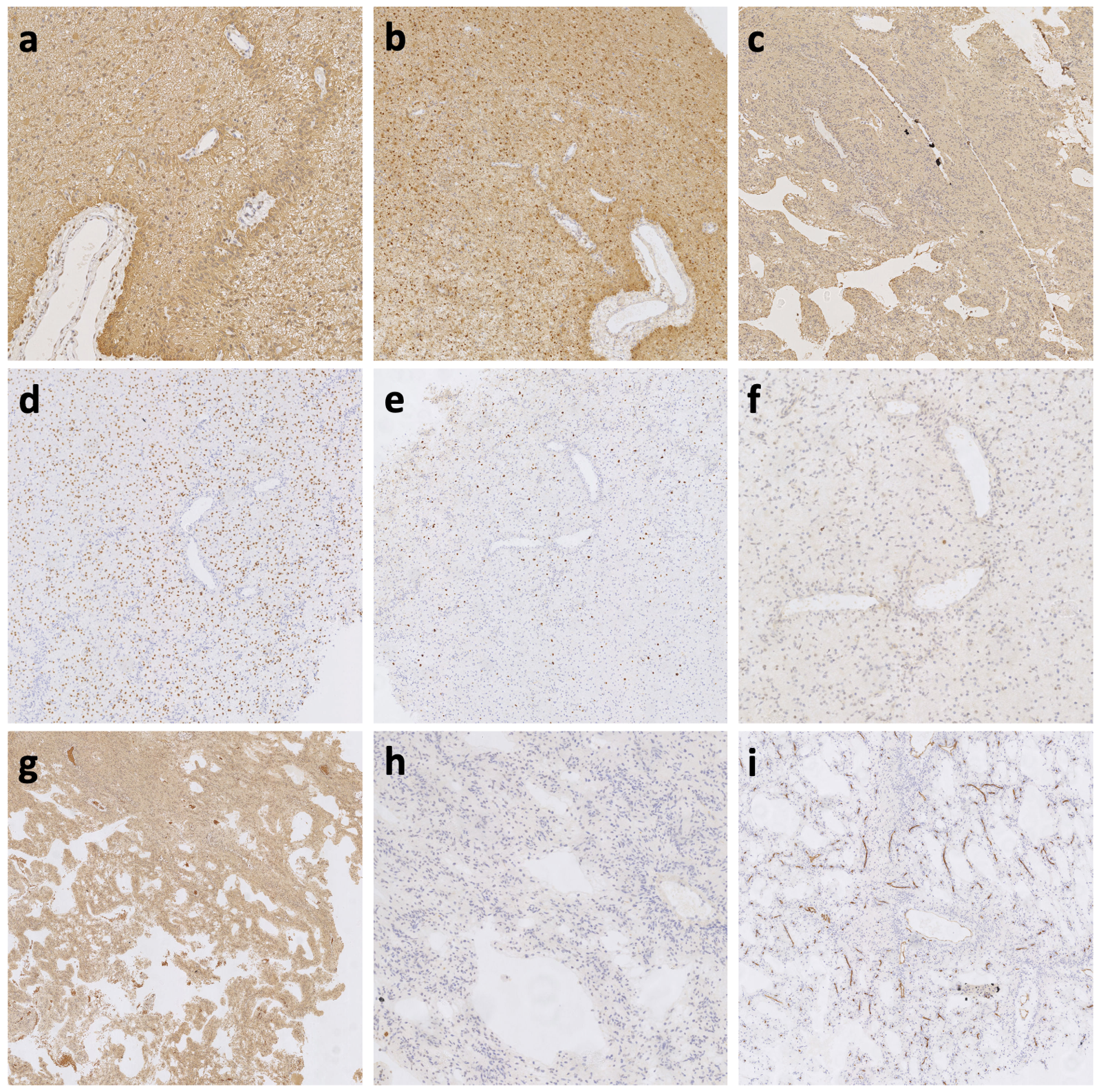


**Supplementary Fig 1.** **Immunohistochemistry of Case 12. a.** GFAP: positive **b.** S-100: positive **c.** Vimentin: positive **d.** Olig-2: partially positive **e.** Ki-67: locally 3~5% **f.** P53: weak positive **g.** BRAF: positive **h.** NeuN: negative **i.** CD34: negative


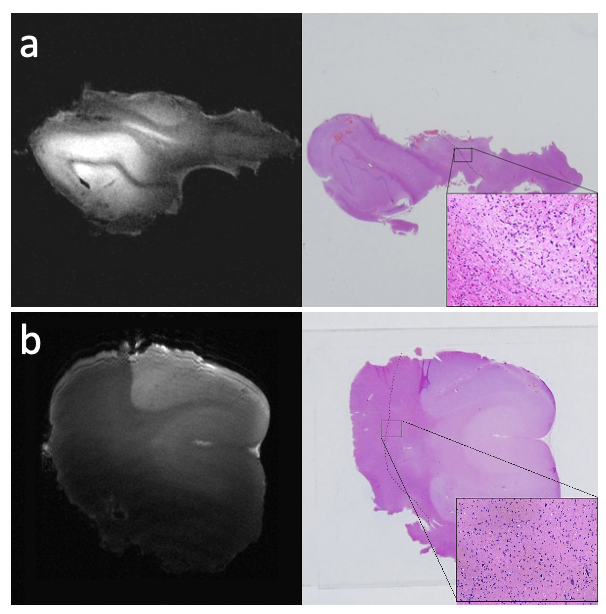


**Supplementary Fig 2. The lesion tissues of two patients compared by MRI and histopathology.** Partial loss of normal cortical structure, abnormal arrangement of cortical nerve cells around the lesions were observed in both cases **a.** Histopathological examination showed slight FCD in temporal lobe and ganglioglioma in hippocampus. **b.** Pathology showed FCD type IIb in the left frontal lobe.


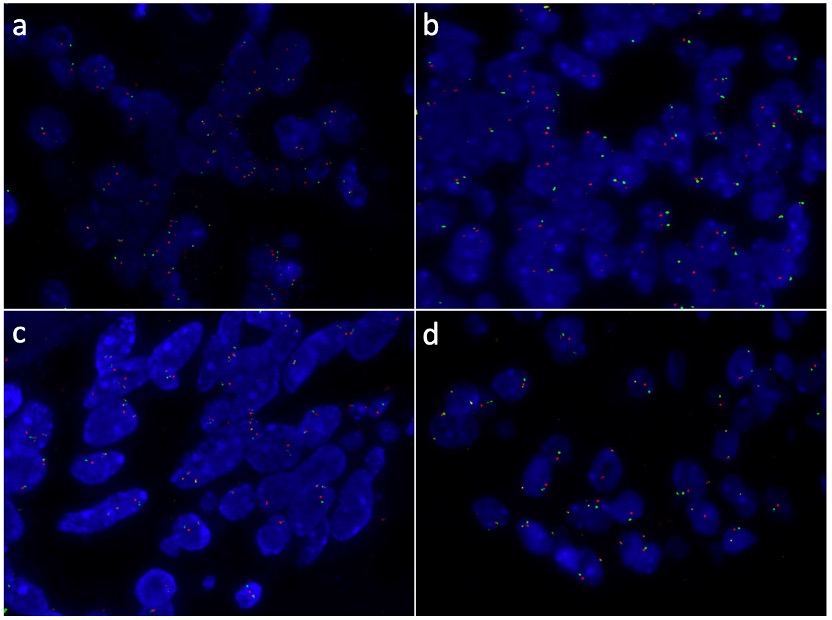


**Supplementary Fig.3 Genetic test results of partial cases. a-b.** No obvious gene fusion in case 5(a) and case 11(b). **c- d.** MYB-QKI fusions could be observed in case 9(c) and case 13(d).
